# Mitral valve aneurysms: echocardiographic characteristics, formation mechanisms, and patient outcomes

**DOI:** 10.3389/fcvm.2023.1233926

**Published:** 2023-08-25

**Authors:** Yi Wang, Shuang Wang, Dandan Chen, Mengmei Li, Sulin Mi, Li Xiong, Wanwan Song, Wei Wang, Shanye Yin, Bin Wang

**Affiliations:** ^1^ Department of Cardiovascular Ultrasound, Zhongnan Hospital of Wuhan University, Wuhan University, Wuhan, China; ^2^ Department of Pathology, Albert Einstein College of Medicine, Bronx, NY, United States

**Keywords:** mitral valve aneurysm, echocardiography, cardiac imaging, infective endocarditis, mitral regurgitation

## Abstract

**Background:**

The accurate etiology of mitral valve aneurysm (MVA) formation is not completely understood, and the most effective management approach for this condition remains controversial.

**Methods:**

We retrospectively analyzed 20 MVA patients who underwent either surgical interventions or conservative follow-ups at the Zhongnan Hospital of Wuhan University between 2017 and 2021. We examined their clinical, echocardiographic, and surgical records and tracked their long-term outcomes.

**Results:**

Of the 20 patients, 12 were diagnosed with MVA using transthoracic echocardiography, seven required additional transesophageal echocardiography for a more definitive diagnosis, and one child was diagnosed during surgery. In all these patients, the MVAs were detected in the anterior mitral leaflet. We found that 15 patients (75%) were associated with infective endocarditis (IE), whereas the remaining patients were associated with bicuspid aortic valve and moderate aortic regurgitation (AR) and mild aortic stenosis (5%), congenital heart disease (5%), elderly calcified valvular disease (5%), mitral valve prolapse (5%), and unknown reasons (5%). Of the 17 patients who underwent hospital surgical interventions, two died due to severe cardiac events. The remaining 15 patients had successful surgeries and were followed up for an average of 13.0 ± 1.8 months. We observed an improvement in their New York Heart Association functional class and mitral regurgitation and AR degrees (*P-*value < 0.001). During follow-up, only one infant had an increased left ventricular end-diastolic diameter and left ventricular end-systolic diameter, whereas the remaining 14 patients had decreased values (*P* < 0.001). In addition, none of the three conservatively managed patients experienced disease progression during the 7–24 months of follow-up.

**Conclusions:**

We recommend using echocardiography as a highly sensitive method for MVA diagnosis. Although most cases are associated with IE or AR, certain cases still require further study to determine their causes. A prompt diagnosis of MVA in patients using echocardiography can aid in its timely management.

## 1. Introduction

**Figure 1:**
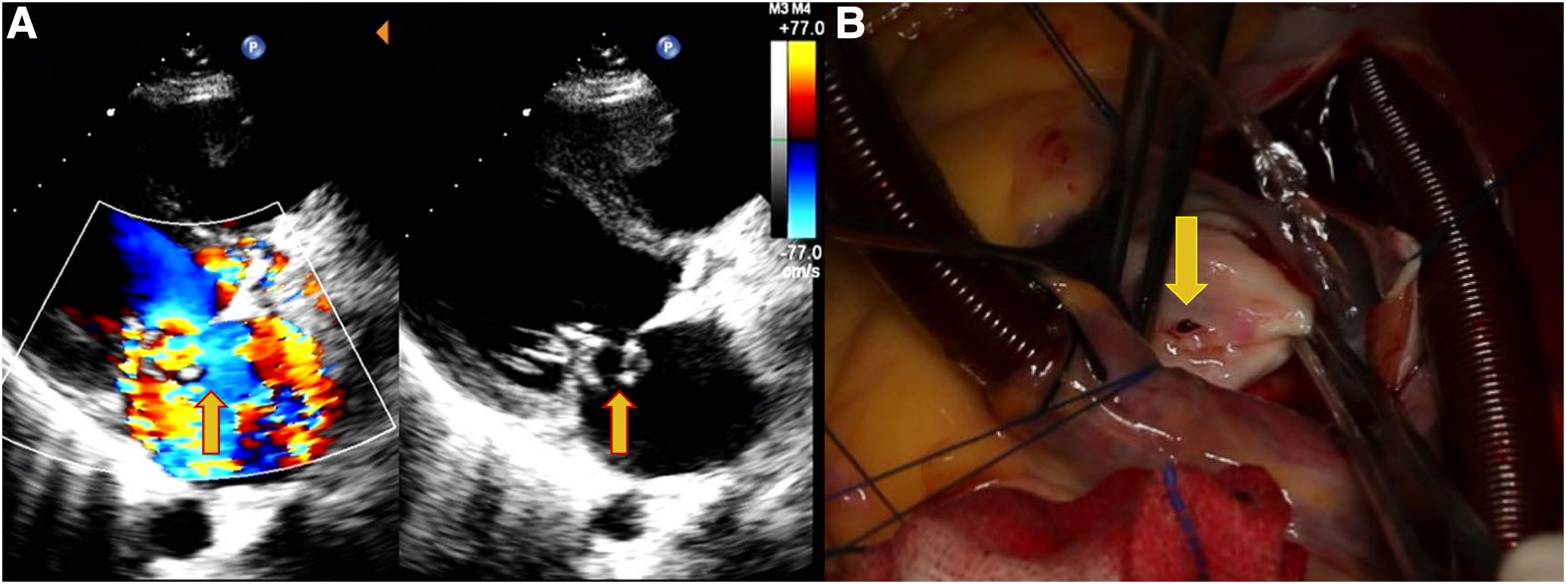
Echocardiography data of a 31-year-old female patient with shortness of breath, palpitations, and fever for 7 days. (**A**) Perforation of the MVA and regurgitation (yellow arrows). (**B**) Site of the MVA perforation during surgery (yellow arrows).

**Figure 2:**
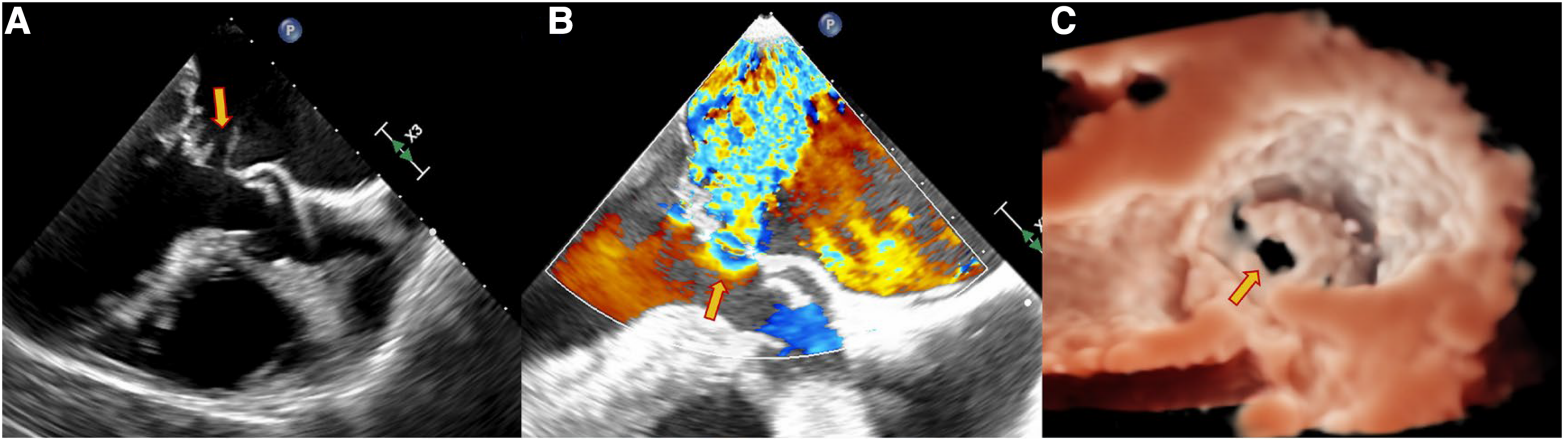
Echocardiography data of a 63-year-old male patient with dyspnea for 4 days. TEE showed the perforation of the MVA as pointed out by the yellow arrow. (**A**) Continuous interruption of the MVA wall. (**B**) Regurgitation from the MVA. (**C**) Continuous breaks on the MVA from a 3D view.

Mitral valve aneurysm (MVA) is a relatively uncommon condition characterized by a localized bulging structure of the mitral leaflet toward the left atrium, exhibiting systolic expansion and diastolic collapse (1, 2), as demonstrated in Figures 1, 2. Despite the rarity of this condition, several case reports and a few series on MVA have been documented. The exact MVA formation mechanisms remain incompletely understood, with infective endocarditis (IE) and aortic regurgitation (AR) being reported as major contributing factors (3–7). Other factors, such as connective tissue defects and valve degenerative changes, have also been implicated (1–3, 5–13). Moreover, cases of congenital mitral valve aneurysms have been rarely reported (14).

MVA management remains a matter of debate due to its potential for significant clinical issues and adverse outcomes. The most common complication observed is aneurysm perforation induced by concurrent mitral regurgitation (MR) (1–8). In addition, MVA has been associated with thromboembolic events (1, 12), increased mitral valve (MV) mean pressure gradient (15), and symptomatic exertional left ventricular (LV) outflow tract obstruction (14). Consequently, selecting an appropriate management approach, whether a surgical intervention involving mitral valve repair or replacement or a conservative follow-up strategy, depends on the presence of ruptured or large aneurysms, severe hemodynamic disturbances, uncontrolled infections, or peripheral embolisms. In patients with uncomplicated conditions, a conservative approach with a close follow-up is often adopted. Echocardiography plays a pivotal role in the accurate MVA diagnosis and treatment decision-making for valvular heart diseases.

In this paper, we present a retrospective analysis of 20 MVA patients, focusing on echocardiographic findings, formation mechanisms, patient management, and outcomes. By integrating anatomical assessments, cardiac function, and hemodynamics, echocardiography offers critical insights into MVA diagnosis and management. We aim to contribute to the understanding of this rare condition and provide valuable insights for clinical practice.

## 2. Methods

### 2.1. Patients

In this retrospective study, we enrolled 20 patients who were diagnosed with MVA in the Zhongnan Hospital of Wuhan University between April 2017 and January 2021. Among these patients, 17 received surgical treatments and three received conservative follow-ups. This study was approved by the Medical Ethics Committee of the Zhongnan Hospital of Wuhan University.

### 2.2. Echocardiography

Transthoracic echocardiography (TTE) examination is commonly performed for the first-line detection and evaluation of MVA. Additional transesophageal echocardiography (TEE) examination was required when TTE could not clearly evaluate the MVA. All diagnostic TTE or TEE examinations were performed using Philips IE 33 (Philips Healthcare, Bothell, WA, USA) with S5-1, X5-1, S8-3, and X8-2t transducers. TTE and TEE examinations were reviewed by the same senior doctor (BW, with 15 years of experience in echocardiography). The diagnostic marker of MVA was a localized bulging structure of the mitral leaflet toward the left atrium with systolic expansion and diastolic collapse.

The diagnostic echocardiographic findings were recorded, namely, MVA location and size, presence of perforation and vegetation, MR and AR degrees, left ventricular end-diastolic diameter (LVEDD), left ventricular end-systolic diameter (LVESD), left ventricular ejection fraction (LVEF), and other abnormalities, such as bicuspid aortic valve (BAV), endocardial cushion defect (ECD), ventricular septal defect (VSD), and patent foramen ovale (PFO). Other clinical information such as clinical manifestations, clinical diagnosis of infective endocarditis, and New York Heart Association (NYHA) functional class was also recorded.

### 2.3. Patient management

The indications for surgical interventions were as follows: (1) patients with acute severe primary MR (16); (2) symptomatic patients with severe chronic primary MR irrespective of the LV systolic function (16); (3) asymptomatic patients with severe chronic primary MR and LV systolic insufficiency (LVEF ≤ 60%, LVESD ≥ 40 mm) (16); and (4) IE patients with heart failure, severe valve dysfunction, and large mobile neoplasms (17). The indications for follow-ups were asymptomatic, LVEF ≥ 60%, LVESD ≤ 40 mm, and none/mild MR and none/mild AR (16). Regular clinical and echocardiography reviews and follow-ups were given to the patients after surgical interventions or those with a conservative follow-up strategy. In addition, the clinical manifestations, follow-up time, NYHA functional class, MR and AR degrees, LVEDD, LVESD, and LVEF for the latest follow-up were collected.

### 2.4. Statistics

Continuous variables were described as average and standard deviation, and categorical variables were described as counts. Two two-sided tests were performed. First, the Wilcoxon signed-rank test was applied to determine the significant differences in the MR and AR degrees between the first diagnosis and the latest follow-up. Second, a paired *t*-test was applied to determine the significant differences in the cardiac systolic function and left ventricular measures between the first diagnosis and the latest follow-up. The significant difference threshold was *P* < 0.05, and the statistical analysis was performed using SPSS 25.0 software.

## 3. Results

### 3.1. Baseline clinical and echocardiographic characteristics of study subjects

Table 1 lists the baseline clinical and echocardiographic characteristics of the 20 patients enrolled in this study, comprising 8 females and 12 males, with a median age of 34 years (range: 7 months–63 years). Among the adult patients, 12 were evaluated using TTE, and seven required additional TEEs for the MVA assessment. Remarkably, the MVA in a 7-month-old child was initially misdiagnosed by TTE, but it was later detected during the surgical intervention for an ECD and a PFO.

**Table 1:** Baseline characteristics and echocardiographic data of the study subjects.

	All patients (*n* = 20)	Surgical interventions (*n* = 17)	Conservative follow-ups (*n* = 3)
Baseline characteristics			
Age, years	36.2 ± 12.4	35.4 ± 11.7	40.0 ± 18.3
Male	12 (60.0)	12 (71.6)	0 (0)
Symptoms^a^	17 (85.0)	17 (100.0)	0 (0)
NYHA functional class			
I	3 (15.0)	0 (0)	3 (100)
II	5 (25.0)	5 (29.4)	0 (0)
III	9 (45.0)	9 (52.9)	0 (0)
IV	3 (15.0)	3 (17.6)	0 (0)
Echocardiographic measurements			
LVEF,%	61.5 ± 5.8	60.1 ± 5.7	67.0 ± 2.0
Infective endocarditis	15 (75.0)	15 (88.2)	0 (0)
With vegetation	12 (60.0)	12 (71.6)	0 (0)
AVE	3 (15.0)	3 (17.6)	0 (0)
MVE	2 (10.0)	2 (11.8)	0 (0)
AVE and MVE	7 (35.0)	7 (41.2)	0 (0)
Without vegetation	3 (15.0)	3 (17.6)	0 (0)
Enlarged LV	16 (80.0)	16 (94.1)	0 (0)
LVEDD, cm	5.82 ± 1.21	6.09 ± 1.10	4.30 ± 0.20
LVESD, cm	3.99 ± 0.98	4.21 ± 0.88	2.72 ± 0.17
Number of MVAs >1	1 (5.0)	1 (5.9)	0 (0)
Diameter range of MVAs, cm	0.5–2.9 × 0.5–2.5	0.5–2.9 × 0.5–2.5	0.5–0.9 × 0.5–0.7
Location of MVA			
A1	1	0	1 (33.3)
A2	5	4	1 (33.3)
A3	2	1	1 (33.3)
A1–A2	5	5	0 (0)
A2–A3	4	4	0 (0)
A1–A3	1	1	0 (0)
Perforation detected	14 (70.0)	13 (76.4)	1 (33.3)
Average maximal diameter, mm	5.35 ± 2.44	5.62 ± 2.33	2
Mitral regurgitation			
None or mild	3 (15.0)	1 (5.9)	2 (67.7)
Moderate or severe	17 (85.0)	16 (94.1)	1 (33.3)
Aortic regurgitation			
None or mild	8 (40.0)	5 (29.4)	3 (100.0)
Moderate or severe	12 (60.0)	12 (70.6)	0 (0)
Other defects beyond valves	6 (30.0)	5 (29.4)	1 (33.3)
In-hospital death after surgeries	2 (10.0)	2 (11.8)	NA

A1/A2/A3, echographical portions of the aortic leaflet; MVE, mitral valve endocarditis.

Values are mean ± SD or *n* (%) unless otherwise stated.

^a^ Symptoms include fever, shortness of breath, fatigue, weight loss, dyspnea, lightheadedness, chest pain, chest oppressive sensation, palpitation, cerebrovascular accident, and a heart murmur.

Of the 19 adult patients, 16 underwent surgical interventions following diagnosis due to clinical symptoms, such as fever, shortness of breath, fatigue, weight loss, dyspnea, dizziness, chest pain, chest tightness, palpitations, and cerebrovascular accidents. These patients presented with enlarged left ventricles, decreased LVEF, and impaired heart function (all NYHA functional class ≥ II). Among them, four had BAVs, three had VSDs, and one had a ruptured right coronary sinus aneurysm in the right ventricle. In contrast, three adult patients received conservative follow-ups for being asymptomatic, and they exhibited normal left ventricles, LVEF, and heart function.

In this cohort of 20 MVA patients, 19 had a single MVA located in the anterior mitral leaflet, involving one or two sub-leaflets of A1–A3. Only one patient had multiple MVAs involving A1, A2, and A3. The size of the MVAs ranged from 0.5 cm × 0.5 cm to 2.9 cm × 2.5 cm. Among the 14 patients with ruptured MVAs in the left atrium, the break size ranged from 0.2 to 1.0 cm, with 11 of them experiencing severe MR, one with moderate-to-severe MR, one with moderate MR, and one with mild-to-moderate MR. The remaining six patients had non-ruptured MVAs, including two with severe MR, one with moderate-to-severe MR, one with moderate MR, one with mild MR, and one with no MR.

Clinically diagnosed IE was present in 15 of the 20 patients (75%). Among these 15 patients, 12 had moderate or greater AR, nine had severe AR, and three had moderate AR. Among the five patients without IE, one had an ECD, one had a bicuspid aortic valve with calcification, one had a bicuspid aortic valve with mild aortic regurgitation and calcification, one had a mitral valve prolapse, and one had gallstones. Further details for each patient are listed in Supplementary Table S1.

### 3.2. Patient outcomes

Among the 17 patients who underwent hospital surgical interventions, two died after surgery due to low cardiac output syndrome or multiorgan failure. However, encouragingly, 15 patients survived and were followed up for 11–18 months (mean follow-up of 13.0 ± 1.8 months). Table 2 lists the postsurgical follow-up data, revealing a significant improvement in the NYHA functional class compared to baseline (*P* < 0.001). Specifically, two patients (13%) had an improvement from NYHA functional class IV to II, eight patients (53%) had an improvement from class III to I, and five patients (34%) had an improvement from class II to I. In addition, there was a remarkable enhancement in the MR and AR degrees, as shown in Tables 3 and 4 (*P* < 0.001).

**Table 2:** Improvements in cardiac function of patients with surgical interventions (*n* = 15).

Pre-NYHA functional class	Post-NYHA functional class				*P*-value
	I	II	III	IV	
I	—	—	—	—	<0.001
II	5 (33.3%)	—	—	—	
III	8 (53.3%)	—	—	—	
IV	—	2 (13.3%)	—	—	

**Table 3:** Improvements in the MR degree of patients with surgical interventions (*n* = 15).

Pre-MR degree	Post-MR degree		*P-*value
	None or mild	Moderate or severe	
None or mild	1 (6.7%)	—	<0.001
Moderate or severe	14 (93.3%)	—	

**Table 4:** Improvements in the AR degree of patients with surgical interventions (*n* = 15).

Pre-AR degree	Post-AR degree		*P*-value
	None or mild	Moderate or severe	
None or mild	—	—	<0.001
Moderate or severe	10 (100%)	—	

**Figure 3:**
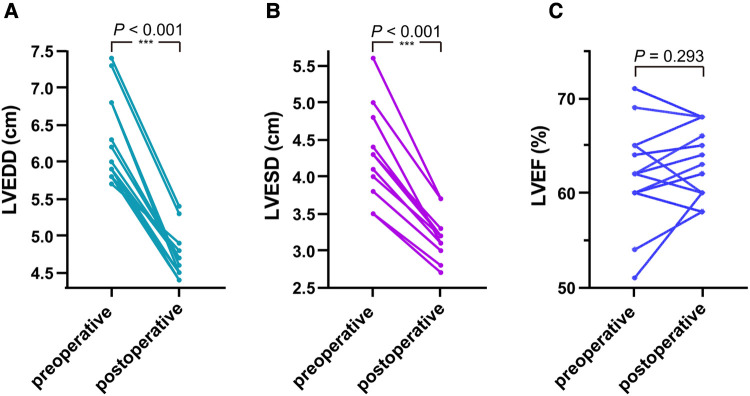
In the 14 adult patients, as shown in (**A**,**B**) comparing the last follow-up measurements with preoperative values, the LVEDD and LVESD were significantly reduced. As shown in (**C**), comparable values were observed on the LVEF.

Analyzing the 14 adults from the cohort (Figures 3A,B), the last follow-up measurements revealed a significant reduction in the LVEDD (6.257 ± 0.580 vs. 4.721 ± 0.304, *P* < 0.001) and LVESD (4.286 ± 0.597 vs. 3.157 ± 0.279, *P* < 0.001) compared to the preoperative values. However, in 6-month-old infants, increased LVEDD (2.4 vs. 2.7) and LVESD (1.7 vs. 1.9) were observed (Figures 3A,B). In assessing the LVEF (%) (Figure 3C), comparable values were noted (61.667 ± 5.038 vs. 62.667 ± 3.697, *P* = 0.293).

In three patients who were managed with a conservative follow-up strategy, the latest follow-up results were available for 7, 8, and 24 months, respectively. Encouragingly, no progression in NYHA functional class or MR and/or AR degrees was observed compared to baseline, and left ventricular measures remained within the normal range throughout the follow-up period.

These results suggest that surgical interventions resulted in significant improvements in NYHA functional class, MR and AR degrees, and favorable alterations in the left ventricular dimensions for the majority of patients. Those managed conservatively also exhibited stable clinical and echocardiographic outcomes during the follow-up period.

## 4. Discussion

Despite significant advancements in antibiotics, critical care, and surgical techniques, MVA remains life-threatening. Early detection and prompt intervention are crucial to prevent complications like rupture and embolism. Echocardiography is the primary screening method for heart vascular diseases and hemodynamic assessment. MVA presents a unique ultrasonic feature of a saccular echo-free space protruding in the left atrium, exhibiting systolic expansion and diastolic collapse. The aneurysm forms on the mitral valve, causing a small disruption in valve continuity known as an orifice that communicates with the left ventricle. Color Doppler flow imaging (CDFI) occasionally reveals blood flow excursions into and out of the MVA during systole and diastole, respectively (3, 18). In our study, we observed this CDFI manifestation in only one patient. The major echocardiographic finding for diagnosing MVA was the visualization of a localized saccular bulge of the mitral leaflet protruding in the left atrium, exhibiting systolic expansion and diastolic collapse, communicating with the left ventricle. Among the patients in our study, 12 were diagnosed with MVAs using TTEs, seven required additional TEEs for further diagnosis, and one infant patient was diagnosed with MVA during surgical intervention for an ECD and a PFO. TEE proved to be a more sensitive tool than TTE in detecting MVA, which is consistent with the previous studies (19, 20). However, misdiagnosis might occur, especially in small hearts, where TTE images may not be clear enough to detect the MVA. Furthermore, MVA has not been previously reported in infants without IE, suggesting a possible association with congenital heart disease.

The MVA formation mechanisms play a crucial role in patient management. The normal mitral valves consist of four layers, and damage to this valvular structure can lead to various lesions, such as prolapse, perforation, and MVA (11). The reported MVA formation mechanisms include direct extension of infection from the aortic valve to the anterior mitral leaflet, impingement of aortic valve regurgitation on the anterior mitral valve leaflet, contact of aortic valve vegetation with the anterior mitral leaflet (kissing lesions), and mitral valve infections (3, 5, 8, 9, 12, 21–27). Aortic valve endocarditis (AVE) is considered the most common cause of MVA (3, 4, 6, 7, 9, 24, 25, 28). In our study, 15 out of 20 patients were clinically diagnosed with infective endocarditis, and among them, 10 had AVE and 12 had moderate or severe aortic regurgitation, consistent with the previous findings. Moreover, MVAs in five patients were not related to endocarditis. In one patient, MVA was associated with BAV and moderate aortic regurgitation. MVA formation in this case might be attributed to the long-term impact of high aortic regurgitation pressure on the anterior mitral valve, leading to structural damage and mitral valve weakness (3, 29). Another case involved a 6-month-old infant with an endocardial cushion defect and a patent foramen ovale. As MVA has not been reported in infants without IE, the MVA, in this case, might be associated with congenital heart disease. The remaining three adult patients had no infective endocarditis or moderate or severe aortic regurgitation. One patient had mild aortic regurgitation with mild aortic valve calcification, another had mitral valve prolapse, and the third had gallstones. These cases suggest that MVA formation can be attributed to various factors, such as elderly calcified vascular disease, mitral valve prolapse, or other isolated vulvitis. All the MVAs in our study were detected on the anterior mitral leaflet, consistent with most previous reports (2, 4, 9, 10, 12, 13, 22, 25, 29, 30). However, some case studies have reported MVAs on the posterior mitral leaflet (31-37), possibly induced by inflammation.

MVA may lead to mitral regurgitation, LV enlargement, and ventricular function deterioration. Among the adult patients in this study, 80% showed varying degrees of LV enlargement and heart failure symptoms (NYHA functional classes II–IV). Surgical treatment was the only effective option for these patients, and most underwent MVR. However, two patients with severe left ventricular enlargement and heart failure symptoms died at the hospital after surgery. The severity of left ventricular enlargement and heart failure might contribute to early postoperative mortality, as observed in previous studies (38). MVR was the preferred approach in most cases due to the association of MVA with other malformations, making valve repair technically challenging. While MV repair had been shown to have better outcomes than MV replacement, as reported in previous studies (4, 39), only a few patients in our study underwent MV repair. Nevertheless, significant improvements in cardiac function and valve hemodynamics were observed during follow-ups for both MV replacement and repair patients. The majority of adult patients exhibited decreased LV size, but in one infant, LV size increased as the heart grew.

## 5. Conclusions

MVA is a rare disease with no distinct clinical manifestations. The majority of MVAs are associated with IE. Echocardiography, particularly TEE, has demonstrated high sensitivity in MVA diagnosis and assessment. In addition, echocardiography enables the evaluation of chamber changes and cardiac function, providing valuable guidance in determining the optimal timing for surgical interventions in MVA patients.

## Data Availability

The original contributions presented in the study are included in the article/Supplementary Material; further inquiries can be directed to the corresponding authors.

## Supplementary material

Supplemental Table S1 Clinical parameters of the patients.
